# Dance Instructors and Early Recognition of Adolescent Idiopathic Scoliosis

**DOI:** 10.5888/pcd23.260460

**Published:** 2026-10-22

**Authors:** Amy Liu

**Affiliations:** 1 Johns Creek High School, Johns Creek, Georgia

Scoliosis is a spinal deformity involving a curvature of the spine that exceeds an angle of 10 degrees (1). Adolescent idiopathic scoliosis (AIS) is a type of scoliosis characterized by an abnormal curvature of the spine that develops during growth. It affects approximately 2% to 3% of adolescents in the general population and is the most common form of scoliosis (2,3). At this time, the etiology of AIS is unknown. Current evidence suggests that genetic predisposition, hormonal factors, nutrition, and environmental influences may contribute to its development (2). AIS is most commonly diagnosed among children, adolescents, and young adults aged 10 to 18 years through school-based screenings or routine pediatric examinations (3). Treatment recommendations vary according to curve severity and skeletal maturity and may include close observation, physical therapy, bracing, or surgery (3).

Three years ago, when I was in eighth grade and 14 years old, I was diagnosed with AIS through a school-based screening program. At the time of the diagnosis, my spinal curvature was measured at 34.5 degrees, which was in the upper range of moderate severity (3). As a result, I was immediately prescribed a full-time brace. As a competitive ballet dancer training since the age of 6 years, I was surprised to learn that I had developed scoliosis to the point of moderate severity. This diagnosis seemed paradoxical considering the extent to which ballet is founded on alignment and postural symmetry, and this experience motivated me to investigate the prevalence of AIS among female ballet dancers.

Researchers have proposed that ballet may either attract people who possess characteristics associated with scoliosis risk or expose dancers to physical demands and nutritional factors that may influence spinal development (4,5). Several studies have reported prevalence rates at more than 20% among female ballet dancers, several times higher than rates observed in the general population (4,5). A study of 75 adult professional female dancers found that 24% of the participants had scoliosis and suggested that hormonal, hereditary, and nutritional factors may have contributed to higher susceptibility among these dancers (4). Similar to what was shown for adult dancers, adolescent dancers had a significantly higher risk of developing scoliosis compared with nondancers in a cross-sectional study, with 30% of the adolescent dancers having a positive test result for scoliosis (5). Although a causal relationship has not been established, the consistency of these findings suggests a possible association between ballet participation among females and AIS. This association suggests that dance communities can serve as a viable setting for scoliosis awareness and early detection.

Currently, AIS is commonly detected through school-based screening programs. In a systematic review of scoliosis detection spanning 448,276 adolescents for the US Preventive Services Task Force, 6 of 7 programs evaluated were conducted in school settings (6). However, school-based scoliosis screenings occur periodically during the first 2 years of the pubertal growth period, which marks the greatest risk of spinal curve progression (7). For young dancers with regular, rigorous training, dance instructors are uniquely positioned to notice signs of scoliosis because they observe students’ posture, alignment, and movement on a regular basis. In many dance studios, including my own, a substantial portion of instruction and training involves evaluating and correcting body positioning. Given this level of observation, dance instructors may be among the first adults to notice asymmetries associated with developing scoliosis.

Despite the opportunity for dance instructors to notice these asymmetries, to my knowledge, scoliosis-related education is not clearly established among dance instructors. Longworth et al highlighted this educational gap by calling for improved scoliosis education among dance instructors and parents after conducting a study of 30 adolescent dancers and 30 age-matched nondancers (5). Without adequate scoliosis education, early warning signs may go unnoticed among adolescents who spend substantial time in dance training. Increasing awareness of AIS among dance instructors could enable them to serve as informed observers who recognize potential indicators of spinal deformity and encourage parents or guardians to seek medical evaluation when concerns arise. Such awareness could contribute to earlier diagnosis and timely intervention, which may reduce the chronic health implications of this condition among adolescents.

The importance of early detection extends beyond physical health. Adolescents with AIS frequently experience psychological challenges, including anxiety, emotional distress, isolation, and concerns about body image (8–10). Adolescence is already a period of physical, emotional, and social vulnerability, and the presence and treatment of scoliosis can further affect self-esteem and social participation (9). In a competitive dance environment, limited awareness among instructors may further exacerbate the mental distress of isolation and alienation among affected adolescents. Research has demonstrated that adolescents with AIS often report greater body image dissatisfaction and psychological distress compared with their peers (8). Heightened awareness along with earlier detection and appropriate treatment may help reduce the emotional challenges associated with AIS. Furthermore, instructors who are informed about the condition and its psychosocial effects may be better equipped to foster a supportive environment, helping affected adolescent dancers feel less isolated and more understood.

One practical approach to addressing this gap would be to incorporate basic scoliosis awareness education into existing training programs for dance instructors. Professional organizations, such as Dance Masters of America, have established training programs for dance instructors in which scoliosis awareness could be integrated (11). Major dance competition organizations, such as the Youth America Grand Prix and the Universal Ballet Competition, attract thousands of dancers each year, bringing together a large population of adolescent dancers and their instructors. From this accessible standpoint, dance organizations could incorporate scoliosis awareness education into existing resources and programs. Such instruction and outreach could focus on common visual indicators of AIS, including uneven shoulders, rib prominence, and asymmetry of the waist or trunk, while emphasizing that instructors are not responsible for diagnosis. Educational materials could be developed through partnerships among dance organizations and pediatric health care providers to ensure that information is professional and accessible. 

Similar to how sports coaches are trained to recognize signs of concussion, dance instructors could be enabled to identify potential warning signs and recommend medical evaluation when appropriate. The National Collegiate Athletic Association, for example, requires member institutions to provide concussion education annually to coaches as part of their concussion safety protocols, with coaches required to acknowledge that they have reviewed and understood the material (12). Similarly, the HEADS UP program at the Centers for Disease Control and Prevention provides training and resources to coaches, sports officials, and school professionals on concussion prevention and education (13). This practice demonstrates how education tailored to a specific condition can be implemented in athletic instruction. These educational initiatives would build on existing training and outreach programs in the dance community, target a population already at elevated risk, and offer a feasible method to promote early detection and mental health for adolescents with AIS.

For these reasons, increasing scoliosis awareness as part of dance education should be considered a potential public health strategy for early detection and timely diagnosis and treatment. Although dance instructors are not health care professionals and should not be expected to diagnose scoliosis, they can play an important role in recognizing potential warning signs and encouraging appropriate medical evaluation. Given the elevated prevalence of AIS among female ballet dancers, educating dance instructors about scoliosis may enhance early detection efforts and improve health outcomes for affected adolescents. Awareness and timely identification can influence treatment opportunities, highlighting the potential benefits of expanding scoliosis education in the dance community. In addition, expanding scoliosis awareness beyond traditional clinical settings leads into a broader public health principle, illustrating how adults who regularly interact with adolescents can play an important role in recognizing health concerns and facilitating timely access to care. My own experience illustrates the current inadequate recognition of AIS among female ballet dancers and the potential for change. With greater awareness and education among dance instructors and dance communities, young dancers with AIS will have better opportunities for care and early detection, and thus better mental and physical health outcomes.
